# Innovation and New Technologies in Spine Surgery, Circa 2020: A Fifty-Year Review

**DOI:** 10.3389/fsurg.2020.575318

**Published:** 2020-11-24

**Authors:** G. Bryan Cornwall, Andrea Davis, William R. Walsh, Ralph J. Mobbs, Alexander Vaccaro

**Affiliations:** ^1^Shiley-Marcos School of Engineering, University of San Diego, San Diego, CA, United States; ^2^Surgical Orthopaedic Research Laboratory, Prince of Wales Hospital, University of New South Wales, Sydney, NSW, Australia; ^3^Bodkin IP, San Diego, CA, United States; ^4^Rothman's Orthopaedic Institute, Thomas Jefferson University, Philadelphia, PA, United States

**Keywords:** spine surgery, innovation, new technology, robotics, artificial intelligence, biologics, spine implants, mini review

## Abstract

Spine surgery (lumbar, cervical, deformity, and entire spine) has increased in volume and improved in outcomes over the past 50 years because of innovations in surgical techniques and introduction of new technologies to improve patient care. Innovation is described as a process to add value or create change in an enterprise's economic or social potential. This mini review will assess two of three assessments of innovation in spine surgery: scientific publications and patents issued. The review of both scientific publications and issued patents is a unique assessment. The third assessment of innovation: regulatory clearances of medical devices and equipment for spine surgery and their evolution over time, will also be discussed.

## Introduction

Improvements and advancements in patient outcomes with spine surgery have been facilitated by many factors including the potential offered by innovations and new technologies. It is necessary to measure outcomes; otherwise, how does one assess whether advancements or benefits are realized? Whether the innovation is in surgical techniques or surgeon training, or efficiencies in surgical care, or the introduction of a new technology, or perhaps new ways to monitor patient outcome, improvements can be derived from process improvements to novel devices.

Innovation is the positive change in process or efficiency that leads to improved value. This may or may not involve an invention or novel new technology. It could be the result of education, introducing a technique or technology from another field, or focusing on other positive metrics and removing inefficiencies or other negative metrics. According to academic business leader and innovation expert, Peter Drucker (1): “At the heart of that activity, entrepreneurship, is innovation: the effort to create purposeful, focused change in an enterprise's economic or social potential.” The same description of innovation could be applied to medicine and the advancement of patient care.

The history of spine surgery is replete with innovators and pioneers. Often in the early phase of introducing an innovation or new technology, these innovators may have been reviled or misunderstood and then over time rejoiced. One such example, Dr. Paul Harrington whose story of developing spinal surgery and implants for children afflicted with polio-induced scoliosis was recently published: “Dogged Persistence” (2) by Dr. Mark Asher. An article published a review of the origins of eponymous instruments for spine surgery, all named for surgeon innovators (3). Other articles reported the innovations and inventions of neurosurgeons and spine surgery (4, 5). Starting in 2001, an annual review of “What's New in Spine Surgery” was summarized in the Journal of Bone and Joint Surgery (6–24). The specific review topics varied each year, but were generally organized into categories of cervical spine, lumbar spine, spinal deformity, biologics in the spine, and occasionally spinal cord injury.

Inventions are the creative process of introducing a new idea which may culminate in a patent. A patent is a contract to protect intellectual property for a period of time in exchange for the public disclosure of said invention (25, 26). Patents however are often only part of the story as other factors influence the translation of an idea into clinical use. These include clinical need, manufacturing cost, reimbursement, and ease of use in solving a problem or improving clinical outcomes.

In 2006 the book: “Emerging Spine Surgery Technologies: Evidence and Framework for Evaluating New Technology” edited by Corbin et al. (27) summarized the emerging technologies in spine surgery of the time (28). Given that innovation is a “focused change,” it is logical that innovation is dynamic and “new” technology is a snapshot of a certain period. For example, Dr. Paul Harrington's ratcheting spinal hooks with rods were new technology in the early 1960's but evolved with measured outcomes, continued innovation, and were ultimately replaced by pedicle screw and rod technology. In “Emerging Spine Surgery Technologies” (27), the book is organized into four sections with the majority of the content covering biologic and tissue engineering and surgical techniques including spinal implants and disc replacements.

While this comprehensive textbook provided a nice overview of emerging technologies of the time, there were no chapters covering lateral surgery, resorbable polymers in spine, additive manufacturing or 3D printing, robotics, artificial intelligence (AI), and machine learning applied to spine surgery. The purpose of this mini review is to assess the trends of innovation in spine surgery over time from 1970 through to 2019, a 50-year period. The mini-review is unique in that it combines both a review of the scientific literature and a review of issued patents as a means to evaluate trends in spine surgery over the past 50 years.

## Materials and Methods

To assess “innovation and new technology” in spine surgery over time, we evaluated two distinct representations of innovation: (1) scientific/clinical interest through peer-reviewed publications, and (2) intellectual property interest through issued United States and European patents. We evaluated the literature in the selected databases over the past 50 years.

### Science/Clinical Publications: Pubmed Database

The “Pubmed” database from the US National Institutes of Health was used to assess the number of peer-reviewed scientific publications associated with a given keyword or combination of keywords. As a representation for how relevant each technology is to spine surgery over time, we evaluated the indexed spine surgery literature using Pubmed as a consistent database. We started with using the keywords: “spine surgery” and assessed the number of publications per year from January 1, 1970 to December 31, 2019. We evaluated the following additional keywords in combination with spine surgery using the logic limiter “AND”: cervical, lumbar, deformity, scoliosis, innovation, new technology, lateral lumbar, resorbable polymer, biologics, disc replacement, image guidance, 3D printing, robotics, and artificial intelligence.

### Intellectual Property (Issued Patents): US and EP Patseer Database

The “PatSeer” database was used to assess the number of granted US and EP (European) patents for each category. The Cooperative Patent Classification (29) and the US Patent Classification (30) were used to identify the different technology areas within the spinal innovation field. Where at all possible, the individual patent subclasses were selected that represent each category by their definition. In some cases where there was not a directly corresponding category or art was classified in further classifications of a more comprehensive nature, additional classification and keyword combinations were used to narrow in on the categories selected: biologics, spinal plates, interbody devices, pedicle screws, image guided surgery, and robotic surgery, all related to spine.

## Results

### Science/Clinical Publications: Pubmed Database

**Figure 1:**
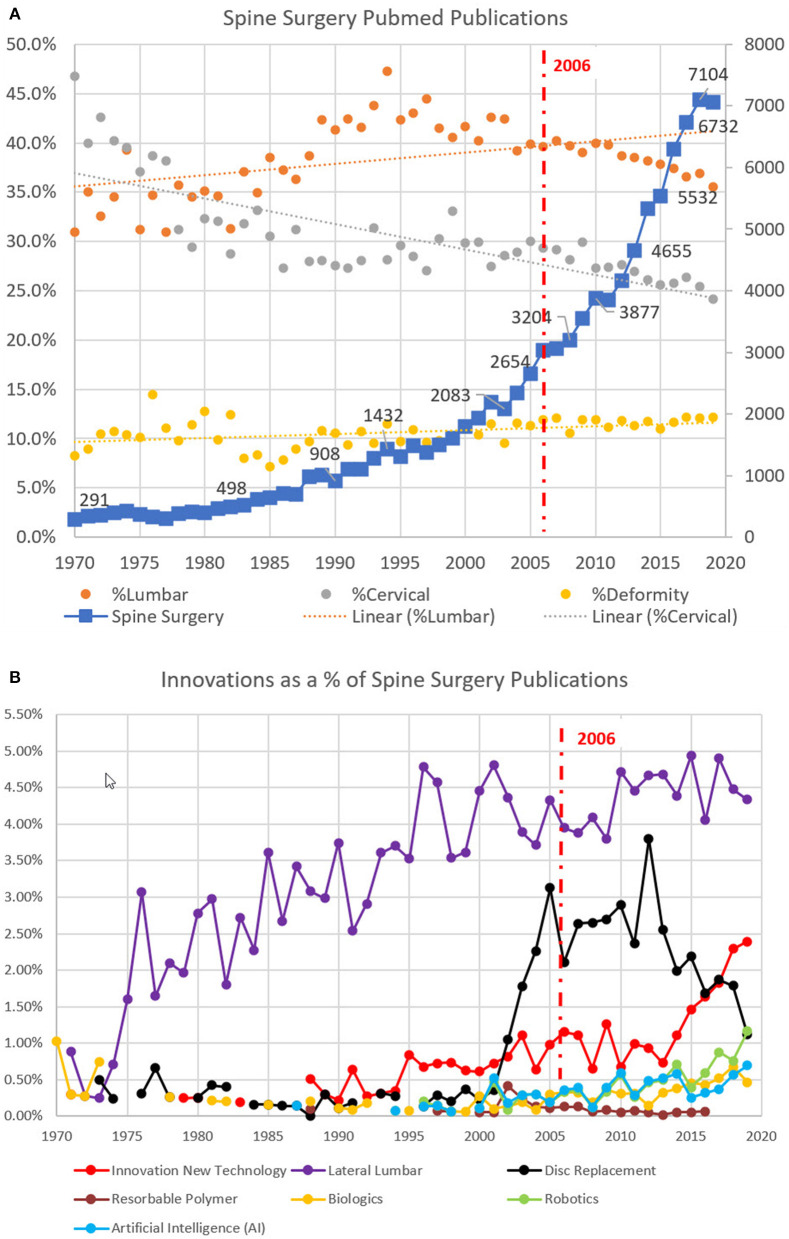
**(A)** Pubmed keyword search for the time (1970–2019) including keywords: spine surgery AND lumbar, cervical, and deformity. **(B)** Pubmed keyword search for the same period (1970–2019) including keywords: lateral lumbar, disc replacement, and resorbable polymer AND spine surgery.

The number of scientific publications with the keyword: “spine surgery,” increased exponentially from 1970 through 2019 as illustrated in Figure 1A. With over 100,000 (103,698) peer-reviewed publications starting with 291 publications in 1970 and with over 7,000 publications in 2019, the growth is demonstrated in the plot of number of publications per year. 65.1% of these publications have occurred since 2006, when the Emerging Technologies in Spine Surgery (27) book was written.

The spine literature was also characterized using the three main classifications identified in the “What's New in Spine Surgery” series of articles: cervical, lumbar, and deformity (or scoliosis). For each keyword AND spine surgery, the subset number of publications was determined per year. The results were expressed as a percentage of the number of spine surgery publications per year as demonstrated in Figure 1A. The trendline for the keyword deformity AND spine surgery was relatively constant across the 50-year period. On average, 11% of the spine surgery literature had the keyword “deformity” with a standard deviation of 1.4% and the proportion of publications ranging from a high of 14.5% to a low of 7.2% for any given year. The trendline of “lumbar” articles has generally increased over the 50-year period with an average of 38.8% of the articles with a standard deviation of 3.7% ranging from a high of 47.3% to a low of 30.9%. The trendline of “cervical” articles has generally decreased over the 50-year period with an average of 27.9% of the articles with a standard deviation of 4.9% ranging from a high of 46.7% to a low of 24.1%.

The number of publications with the various innovation and new technology keywords AND spine surgery are summarized in Table 1. These keywords were also assessed as a percentage of the spine surgery literature over time and plotted in Figure 1B. Starting with the keywords: innovation OR new technology AND spine surgery, there were 1,162 publications between 1970 and 2019 with 83% of those articles published after 2006. One technology in the book (27), disc replacements in spine surgery, were featured in seven book chapters. Using the keywords: disc replacement AND spine surgery, there were 1,723 publications between 1970 and 2019 with 85% of those articles published after 2006.

**Table 1:** Categories or Technologies and keyword “Spine Surgery.”

**Keyword**	**# Publications (1970–2019)**	**% of Spine Surgery**	**# Publications (2006–2019)**	**% of Total Spine Surgery (1970–2019)**	**# Years with Keyword**
Spine Surgery	103,698	100.0%	67,474	65.1%	50
Cervical	28,927	27.9%	17,987	62.2%	50
Deformity	11,568	11.2%	7,861	68.0%	50
Lumbar	40,263	38.8%	25,666	63.7%	50
Innovation or New Technology	1,162	1.1%	970	83.5%	36
Artificial Intelligence (AI)	339	0.3%	298	87.9%	25
Biologics	319	0.3%	272	85.3%	36
Disc Replacement	1,723	1.7%	1,471	85.4%	41
Image Guidance	2,331	2.2%	1,911	82.0%	37
Lateral Lumbar	4,257	4.1%	2,988	70.2%	49
Resorbable Polymer	56	0.05%	31	55.4%	20
Robotics	441	0.4%	403	91.4%	24
3D Printing	190	0.18%	190	100.0%	7

Two examples of new technologies that did not appear in the 2006 Emerging Technologies book (27): resorbable polymers for spine surgery and lateral lumbar spine surgery. Using a similar keyword strategy there were 56 publications with resorbable polymers AND spine surgery with 55.4% of those publications after 2006 and there were 4,257 articles concerning lateral lumbar AND spine surgery with 70% of those published after 2006. Clearly lateral spine surgery has continued to be a relevant innovation while resorbable polymers has not been a new technology that has stood the test of time.

### Intellectual Property (Issued Patents): US and EP Patseer Database

**Figure 2:**
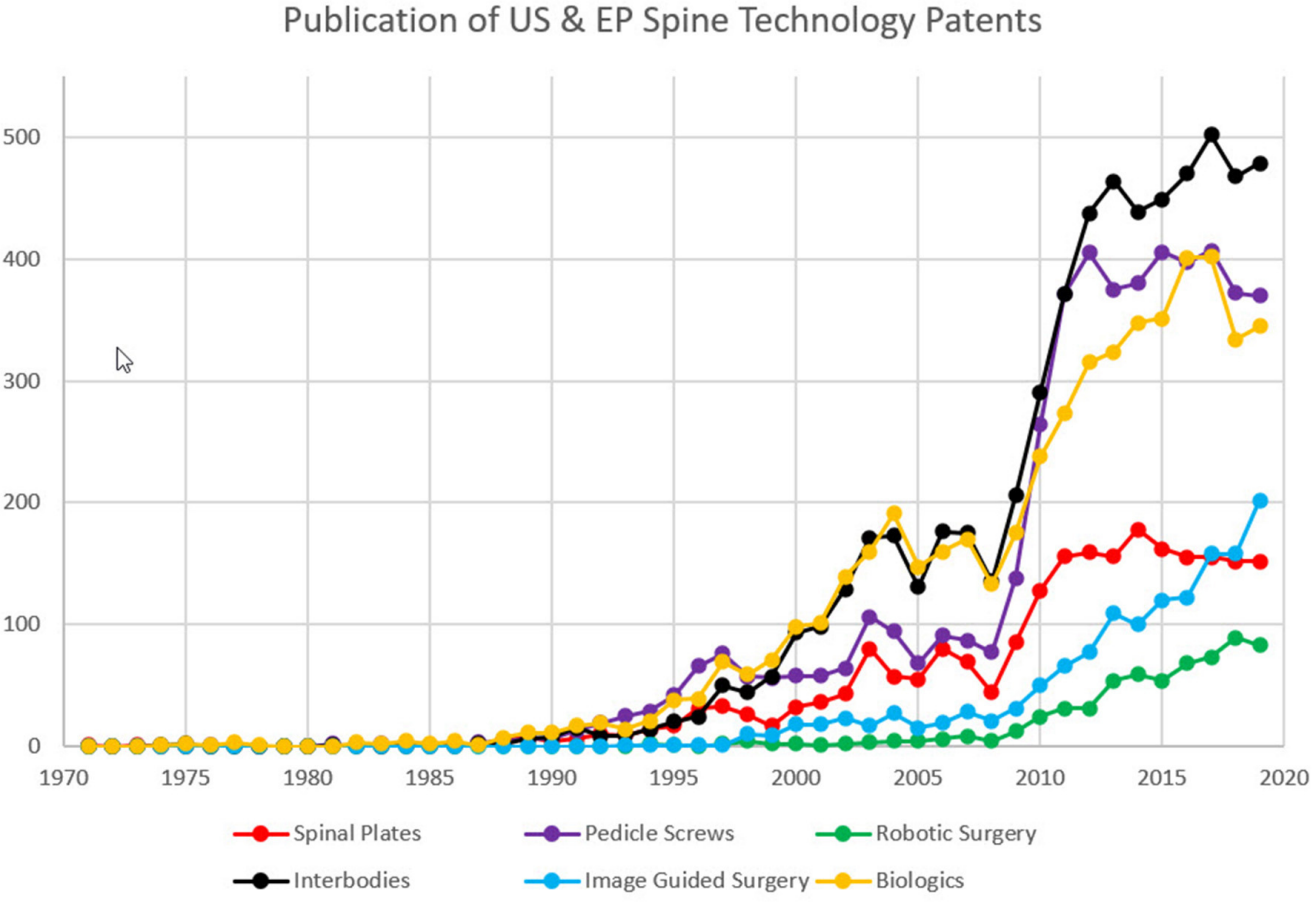
Intellectual Property assessment over time with number of US and EP patents issued for the period (1970–2019).

The entire dataset of 16,336 records of patent grants from January 1, 1970 to December 31, 2019 was analyzed and plotted in Figure 2 using the patent analytics software. Publication trends of US and EP granted patents with time are summarized in Figure 2. In the first two decades, between 1970 and 1990, there was almost no patenting activity in this domain with the categories having mostly none, but at most 10 granted patents each year. Spinal patenting trends for the mechanical technologies (pedicle screws, spinal plates, and interbodies) and biologics slowly start to grow post 1990. All of them seem to have a decrease in 2008, possibly due to the recession of that time, then increase exponentially from that point on for the following 4 years. Starting 2014 the graphical representation shows a plateau with 450–500 patents/year related to interbodies, 150/year related to spinal plates (including cervical, thoracic, and lumbar), 350–400 patents/year related to pedicle screws, and about the same for biologics. The more modern categories of image-guided and robotic surgery have seen a steady increase from about 2008.

The patenting trends have to be seen as a delayed innovation proof in view of the quite lengthy average time to get a patent, which between 2008 and 2015 was about 3.5 years in the US and 5.5 years at the European Patent Office (31).

## Discussion

In most of the industrialized world, the metrics associated with spine care are improving (32). More patients are having surgery and those patients are getting better with care. Is this the result of innovation or invention? Companies and even health care systems are perceiving the benefit of innovation largely driven by the greater business community and the focus on innovation across many industries (1).

We were interested in using regulatory clearances as another reflection of interest in innovation and introduction of new technologies over time. However, this investigation proved to be problematic for numerous reasons. First, there is no global database for the regulatory clearance or approval of medical devices. While scientific literature does have global databases such as Pubmed, regulatory approvals tend to be more regional or country specific. Even in a large market covering the European Union, there is currently no medical device database (33, 34). In the largest spine market, the USA, there are three different databases that could be assessed but they cover different time periods and have different relative pros and cons (35). The first is the US Food and Drug Administration (FDA) premarket notification or 510(k) database for devices that are cleared based upon achieving substantial equivalence to another cleared device. The data goes back to 1976 when medical devices were added to the amended federal food, drug, and cosmetic (FD&C) act. The majority of spine devices introduced to the market fall under the 510(k) pathway (35). The second database is for devices that fall under the premarket announcement (PMA) requiring a clinical study prior to regulatory approval. New technologies such as total disc replacements for both lumbar and cervical fall under this pathway. A third database to investigate is the National Institutes of Health (NIH) US National Library of Medicine ClinicalTrials.gov database of registered clinical trials. There were 784 studies registered with the keywords spine surgery. The assessment of regulatory clearances could provide additional clues about innovation and new technologies introduced into the marketplace. However, the authors considered the disparity of information between these regulatory databases to limit the usefulness of this information in this mini review.

In the 2001 article, “What's New in Spine Surgery” (6), the authors commented that in the last 20 years from 1981 to 2001, the number of spine fellowship programs had increased from <15 to more than 200. In 2006, the book on Emerging Technologies in Spine (27, 28) described an innovative time and a period of prolific introduction of new technologies hoping to improve patient care. As more and more technologies were introduced, there was more push-back from regulatory bodies in both the United States (US) and in the European Union (EU) to improve the burden of proof and from insurance providers adding more scrutiny to what technologies would be reimbursed (or paid for).

Patenting trends are strongly influenced by legal and political measures taken in the jurisdiction of filing. Supreme Court decisions, instituting new measures like the PTAB (US Patent and Trials Board) and AIA (Leahy–Smith America Invents Act) all contribute to changes in the big medical device companies' patent strategies. As seen in Figure 2, all the mechanical categories and biologics seem to have a decrease in 2008, possibly due to the recession of that time, then increase exponentially from that point on for the following 4 years. The sharp increase may be in part influenced by the Affordable Care Act signed into law in 2010 and the medical device tax that became effective January 2013 (36).

Another new technology and innovation that was not considered in 2006 was the application of wearable technology sensors (37). Recent work has evaluated the use of “wearables” for objective measurements for outcomes analysis (38). Most clinical studies employ subjective observations to evaluate clinical outcomes. The use of inertial markers or other motion tracking technology to assess objective data could be a potential innovation to improve patient care.

The effect of surgeon training as an innovation in process was not evaluated in detail in this mini-review. Surgeon training can have positive influences on patient outcomes and there has been interesting research performed in this area (39–41). The effect of training was thought to be difficult to evaluate with both a scientific and intellectual property perspective. There has also been interesting research conducted utilizing new technologies such as 3D printing (42) and virtual reality (43, 44). Hopefully, these new innovations in training with new technologies will translate into improved patient outcomes.

Aging population and technological developments will likely drive innovation in the field of spinal surgery. Growth in patenting is expected in areas that have evolved in the past few years, such as spinal navigation, robotic surgery, minimally invasive surgery, patient-specific implants, and 3D printing.

In 2020, there continues to be an interest in both innovation and in new technologies for spine surgery to improve spine care and clinical outcomes. Innovation has been described as a process to add value or create change in an enterprise's economic or social potential. As the global pandemic associated with Covid-19 continues to unfold, innovation will continue to be necessary for all constituents: patients, surgeons, hospitals, health-care providers, industry, payors, and governments.

## Author Contributions

Study concept: GC, WW, and AV. Data collection: GC and AD. Data analysis: All authors. First draft: GC. Review and editing: All authors.

## Conflict of Interest

GC, WW, RM, and AV are all employed by academic institutions and provide consulting services to spinal device companies. AD was employed by the company Bodkin IP. AD is a patent consultant and her business surrounds Intellectual Property research. There was no compensation for any authors in writing this manuscript. All authors declare that the research was conducted in the absence of any commercial or financial relationships that could be construed as a potential conflict of interest.
